# Econometric methodology for selecting explanatory factors for power consumption

**DOI:** 10.1016/j.mex.2025.103601

**Published:** 2025-09-05

**Authors:** Serge Guefano

**Affiliations:** University of Douala, University Institute of Technology, Laboratory of Technologies and applied science, P.O. BOX 8698, Douala, Cameroon

**Keywords:** Causality, Electricity consumption, Explanatory factors, Cameroon

## Abstract

A thorough and careful choice of inputs is the bedrock of all successful energy demand modelling. However, the choice of inputs in most research into fluctuations in electricity demand and its potential determinants is very often made by establishing a simple correlation or by a short descriptive analysis of the specific characteristics of each selected input. As a result, this work proposes a new methodology for input selection based on the interaction between stationarity tests, the Johansen cointegration test, as well as ARDL and VECM modelling, in order to facilitate the highlighting of a real causal link between the inputs brought in the modelling of electricity consumption. Applying this new approach to the Cameroonian case, enables to establish the existence of a unidirectional causal relationship from GDP per capita, CO2 emissions, urbanization and the number of subscribers, respectively, to residential electricity consumption. These inputs are therefore the main factors explaining electricity consumption in this sector.

• A new methodology for selecting input variables in modelling power demand is outlined.
• The stationarity and Johansen tests as well as the ARDL and VECM models are combined.
• The choice of inputs for modelling and forecasting power demand is optimized.

## Graphical abstract

**Figure:**
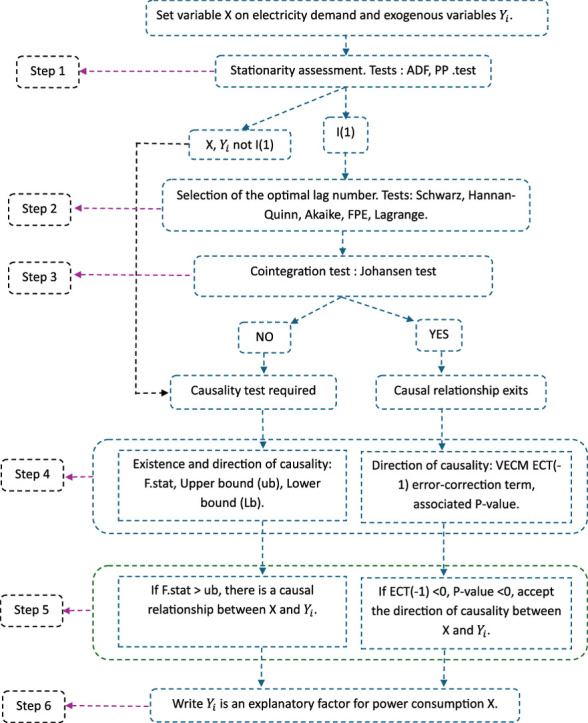

**Specifications table**

**Table:** 

**Subject area**	Energy
**More specific subject area**	Electricity; Renewable energy; modeling; Forecasting.
**Name of your method**	Econometrical methodology for designing electricity consumption factors
**Name and reference of original method**	1- [1] CHONTANAWAT, Jaruwan. Dynamic modelling of causal relationship between energy consumption, CO2 emission, and economic growth in SE Asian countries. *Energies*, 2020, vol. 13, no 24, p. 6664. https://doi.org/10.3390/en13246664 2- [2] GUEFANO, Serge, BOZORG, Mokhtar, CARPITA, Mauro, et al. Causality between residential electricity consumption and explanatory factors. *Energy Strategy Reviews*, 2023, vol. 49, p. 101,155. https://doi.org/10.1016/j.esr.2023.101155
**Resource availability**	https://data.mendeley.com/datasets/b7xtffwy4t/1]

## Background

**Fig. 1:**
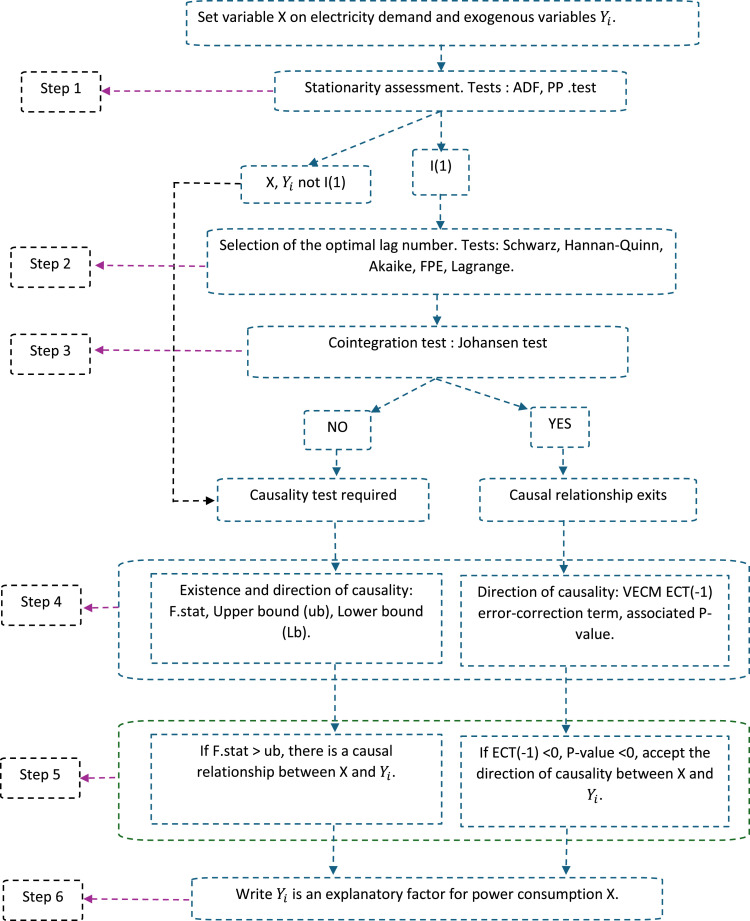
Program to identify explanatory factors for power consumption through causal relationship analysis.

Highlighting formal causal relationships between energy consumption and various explanatory factors is crucial to the success of modelling and forecasting applications. Accordingly, in 2020, Jaruwan [1] proposed a method to facilitate the analysis of cointegration and causality between inputs to time-series models. In the procedure described, a combination of stationarity tests, Johansen's cointegration test, Granger causality analysis in VAR (Vector autoregressive model) or VECM (Vector error correction model) modelling is proposed. However, Johansen's cointegration test is limited when the input variables considered are of a small size (*n* ≤ 30 observations) [2]. Consequently, this work proposes a methodology that includes upper and lower bounds tests in the previous combination whose cointegration test results are more accurate when inputs are of a small size. This technique enables selection of the most significant input variables for modelling electricity consumption in Cameroon's residential sector [2]. In 2020, Jaruwan et al. [1] proposed a three-step econometric approach to improve the accuracy of optimal input selection in energy demand modeling (Fig. 1):

1. Assessment of the stationarity of each variable. If all variables are I(1), proceed to step 2. Otherwise, if at least one variable is non-I(1), proceed to step 3a.
2. Assess cointegration between variables using Johansen's method. If the evaluated cointegration is positive and significant, proceed to step 3b
3. Estimate causality on Granger frameworks.
3.a. Testing the existence and direction of causality using the vector autoregressive (VAR) model.
3.b. A long-run relationship exists so there must be causality for at least one direction. In this case, evaluate the direction of causality using the vector error-correction model (VECM). We then distinguish between two types of causality: short-run causality and long-run causality.

If the tests in steps 1 to 3 are positive and significant, then the selected inputs can be retained as the most relevant variables for the proposed modeling. However, the Johansen cointegration test indicated in step 2 can only provide significant results when the input observations have a length n greater than 30 observations. To overcome this limitation, this paper proposes a methodology that incorporates the Autoregressive Distributed Lag (ARDL) technique to evaluate with greater precision the cointegration of inputs, whether or not the length exceeds 30 observations.

The remainder of this paper is organized into three main sections. Details of the proposed econometric methodology, the validation method and the inherent limitations of the approach highlighted.

## Method details

In this section, the steps necessary for the sequential selection of explanatory factors for electricity consumption are described. These include stationarity tests, criteria for selecting the optimal number of lags of the input variables, the Johansen test for determining the order of long-term cointegration of the selected inputs, ARDL modelling for verifying the results of the Johansen test when the input variables have a length *n* ≤ 30 consecutive observations, and the determination of the existence of a causal relationship by means of upper and lower bound tests. VECM modelling is also described in the context of highlighting the direction of the causal relationship between input variables over the short and long term respectively. Finally, diagnostic test procedures are described to confirm the statistical accuracy of VECM modelling.

### Stationarity tests process

The raw data for most time series variables are non-stationary time series [2]. To avoid any biased causality analysis, it is necessary to ensure their stationary properties [3]. To do this, two statistical tests can be used. The Augmented Dickey Fuller (ADF) test, and the Phillips-Perron (PP) test [3]. Indeed, ADF tests rely on the ordinary least squares method to assess the non-stationarity of an input variable, and the transformation into its stationary form where appropriate. Three approaches are used for this purpose. Firstly, stationarity is assessed on the assumption that the observations present neither a constant nor a trend. This situation is modelled by the relationship Eq. (1). Secondly, the ADF test is performed on the assumption that observations of the input variable under consideration exhibit a constant. This assumption is modelled by relation Eq. (2). Thirdly, stationarity is assessed on the assumption that the input under study has a trend and a constant. This last evaluation point is expressed by equation Eq. (3). In the most cases, these three procedures converge. The results are almost identical, with a few differences appearing at the hundredths level. However, it is necessary to carry out all three test approaches in order to be sure of the stationary or non-stationary nature of the input variable under study.

(1) $$\Delta w_{t}=\xi w_{t-1}+\sum_{i=2}^{q}\lambda_{i}\Delta w_{t-i+1}+\upsilon_{t\;}.$$

(2) $$\Delta w_{t}=\xi w_{t-1}+\sum_{i=2}^{q}\lambda_{i}\Delta w_{t-i+1}+c+\upsilon_{t\;}.$$

(3) $$\Delta w_{t}=\xi w_{t-1}+\sum_{i=2}^{q}\lambda_{i}\Delta w_{t-i+1}+bt+c+\upsilon_{t\;}.$$

In these equations, t is the time index, c is an intercept constant called a drift, b is the coefficient on a time trend, ξ is the coefficient presenting process root, i.e. the focus of testing, q is the lag order of the first-differences autoregressive process, $\upsilon_{t\;}$ is an independent identically distributes residual term. The difference between the three equations concerns the presence of the deterministic elements c (a drift term) and bt (a linear time trend) [4].

These equations represent autoregressive models of order 1 with a constant and a trend, respectively. The specification error $\upsilon_{t\;}$ is assumed to be a process of white noise [5]. Machine execution results highlight a probability (P-value) at which the test performed is significant [1]. Generally, this probability is compared to the critical threshold α for rejecting or validating test results. Thus, if P−value<αwe accept the null hypothesis H***_0_***_:_ there is at least one-unit root. The process is said to be stationary at the critical threshold α. Otherwise, if P−value>α, the null hypothesis is rejected: there is no unit root. The process is non-stationary. As a result, the stationarity of these chronological series must be determined using the sequence of first differences [6]. The results of the ADF tests are reinforced by the unit root tests developed by Philipps-Perron (PP) [7]. Indeed, ADF tests do not consider any heteroscedasticity in the error term but assume that the errors within the model are independent of each other and constitute white noise. The unit root test developed by Philipps-Perron (PP) overcomes the shortcomings of ADF tests and highlights the existence of a unit root more precisely. Constructed to take heteroskedastic errors into account, the test (PP) is subdivided into four steps structured as follows [8]:

- Ordinary least squares estimation of the three basic models of the Dickey-Fuller tests and calculation of the associated statistics and the residual.
- Short term variance estimation: ${\hat{\sigma }}^{2}=\frac{1}{n}\sum_{t=1}^{n}e_{t}^{2}$
- Estimation of the corrective factor $s_{t}^{2}$called long term variance and defined by Eq. (4).
  (4) $$s_{t}^{2}=\frac{1}{n}\sum_{t=1}^{n}e_{t}^{2}+2\sum_{i=1}^{l}(1-\frac{i}{l+1})\left[\frac{1}{n}\sum_{t=i+1}^{n}e_{t}e_{t-i}\right]$$

where l is the number of lags defined by the Newey-West truncation as a function of the number of observations n: $l\approx 4{\left(n/100\right)}^{2/9}$;

- Calculation of pp statistics by relation Eq. (5).
  (5) $$pp=\sqrt{v}*\frac{\left(\hat{\phi }-1\right)}{{\hat{\sigma }}_{\phi }}+\frac{n\left(v-1\right){\hat{\sigma }}_{\phi }}{\sqrt{v}}$$

with $v=\frac{{\hat{\sigma }}^{2}}{s_{t}^{2}}$ which takes the value 1 if $e_{t}$ is white noise.

- **The optimal lag number**

This refers to the time lag j retained simultaneously by most of the chosen tests and capable of tracing the endogenous variable's trends as accurately as possible [9]. The Schwarz (Sc), Hannan-Quinn (HQ), Akaike (AIC) and Lagrange criteria are used to numerically select the right number of lags of the input variables.

- **The Johansen cointegration test process**

The Johansen test is a procedure for testing cointegration of several, say δ, I(1) time series. This test permits more than one cointegrating relationship [10]. There are two types of Johansen test, either with trace or with eigenvalue, and the inferences might be a little bit different. The null hypothesis for the trace test is that the number of cointegration vectors is $\omega =\omega^{*}<\delta \;$. Testing proceeds sequentially for $\omega^{*}=1,\;2,\;\ldots ,\;$and the first non-rejection of the null is taken as an estimate of ω [10]. The null hypothesis for the maximum eigenvalue test is as for the trace test but the alternative is $\omega =\omega^{*}+1$ and, again, testing proceeds sequentially for $\omega^{*}=1,\;2,\;\ldots ,$ with the first non-rejection used as an estimator for ω [10]. Once the stationarity of all input variables in the same order has been established, the Johansen cointegration test is performed using trace statistics $\left(\lambda_{trace}\right)$and eigenvalue statistics $\left(\lambda_{\max}\right)$ [11]. At the 5░%tolerance level, each of these statistics is compared to its number-based critical value. The possible orders of cointegration that may exist between the inputs are tested according to the values of the number ω∈N. The accepted order of cointegration is the one in which the values of $\left(\lambda_{trace}\right)$and $\left(\lambda_{\max}\right)$are less than the critical values and significant at the 5░%level [11].

- **ARDL approach for validating a long-run equilibrium**

In order to validate the hypothesis of a long-run equilibrium between the variables, in addition to the Johansen test, this study uses ARDL modeling and upper-bound tests based on F-statistics. The ARDL technique has the advantage of being more efficient in the case of small and finite samples. In addition, the application of this technique makes it possible to obtain unbiased long-run estimates. According to Barkhordari [12], the F-statistic is compared with the lower bound (Lb) and upper bound (Ub), respectively. If F-statistic > Ub, the hypothesis of non-cointegration is rejected. If F-statistic < Lb, the null hypothesis of non-cointegration cannot be rejected. Nevertheless, if Lb < *F*-statistic < Ub, the null hypothesis of no cointegration is not conclusive.

- **Vector Error Correction Model**

For a general VAR(p) model: There are two possible specifications for error correction: that is, two vector error correction models (VECM): the long run VECM and the transitory VECM [13]. The existence of cointegration means that there is at least one equilibrium relationship between the variables. According to Engel and Granger [14], the existence of an integral vector between the variables suggests that there is a causal relationship between them, at least in one direction. Since the variables are integrated, we can continue the estimation of the error correction model that integrates short-term dynamics with long-term equilibrium [15,16]. The VECM makes it possible to detect the direction of causality in the long run, under the condition of stationarity of the different variables in first difference [16]. In this context, the error-correction term in the model must have a negative sign and be significant at the tolerance threshold [17]. Furthermore, the Wald causality test proves to be significant for assessing the direction of short-term causality, given the significance of the nullity test for the coefficients of the specified VECM model [18].

In general, endogenous and exogenous variables must have a similar trend, so that when considered simultaneously within the model, the response to mutual interactions is negligible, and the only effect is on the designated endogenous variable. This check is necessary at the initialization step of the considered inputs.

**Fig. 2:**
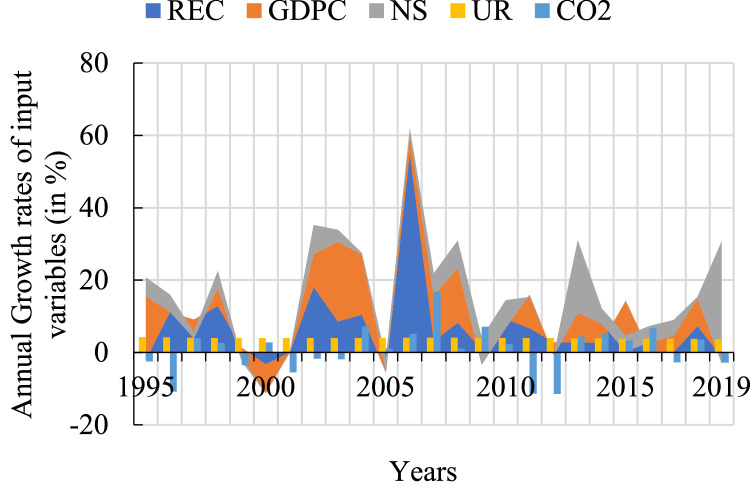
Annual growth rates of variables.

It may turn out that this preliminary text is limited, and that the input variables under observation show only weakly convergent trends. In this case, it is necessary to have an overview of the growth rates of the variables involved. The variables must show quasi-similar growth rates to be considered in the VECM model. This constraint is verified in article [2]. The variables selected show a quasi-similar growth rate, as shown in Fig. 2.

In the specific case where electricity consumption in the residential sector is the objective variable, the associated VECM is defined by the equation Eq. (4).

(6) $$\Delta {\left(REC\right)}_{t}=\alpha \;+\;\sum_{i=1}^{l}\beta_{i}\Delta {\left(REC\right)}_{t-i\;}+\sum_{i=1}^{m}\gamma_{i}\Delta {\left(GDPC\right)}_{t-i\;}+\sum_{i=1}^{n}\lambda_{i}\Delta {\left(CO_{2}\right)}_{t-i\;}+\sum_{i=1}^{P}\psi_{i}\Delta {\left(NS\right)}_{t-i\;}+\sum_{i=1}^{s}\eta_{i}\Delta {\left(UR\right)}_{t-i\;}\;+\;\phi_{j}ECT_{r,t-j}\;+\;\varepsilon_{t}$$

In this equation, the white noise error term is $\varepsilon_{t}$, the error correction term is $ECT_{r,t-j}$ where r corresponds to the accepted order of cointegration. $\phi_{j}$ Denote the adjustment coefficients used to access the level of imbalance correction within the model. αIs the intercept, l,m,n,p,ands are lag numbers. $\beta_{i}$, $\gamma_{i}$, $\lambda_{i}$, $\psi_{i}$, and $\eta_{i}$ are the estimated coefficients, Δ is the symbol for the first difference, which gives the underlying variable its stationary property.

## Method validation

Diagnostic tests are used to validate the statistical consistency of the specified VECM and its relevance to the study of causal relationships. These include tests for Ramsey Reset specification, heteroskedasticity, normality of error terms and overall model stability [[19], [20], [21]].

- Ramsey reset test

To ensure that the explanatory factors selected are sufficient to model electricity consumption, we carry out the Ramsey specification error test. The P values associated with the t-statistic, the F-statistic and the likelihood ratio of the test must be, respectively, greater than the tolerance threshold set at [22].

- Heteroscedasticity test

When the variance of the error term within the model is not constant, the specified model is subject to heteroskedasticity [23]. This may be due to several statistical inconsistencies including:

• The repetition of the same value of the variable to be explained for different explanatory variable values.
• The relationship between errors and the values of an explanatory variable.
• The observations are averages calculated from various sample sizes.

Such circumstances may skew the quality of the results, resulting in incorrect analyses and conclusions. Several tests can be used to detect and correct heteroscedasticity in a time series. A Lagrange test, for example, can be carried out by computing the statistic $LM\;=\;n^{\prime }*R^{2}$ (n’= *n*-p: difference between the number of observations n and the order of autocorrelation p) [20]. $R^{2}$is the associated determination coefficient, which describes the averaging of the series' values). Then we compare $\chi_{\alpha }^{2}\left(p\right)$ to the αthreshold in the order p. If $LM\;=\;n^{\prime }*R^{2}>\chi_{\alpha }^{2}\left(p\right),$ the hypothesis of error independence is rejected; in other words, the F-statistic and $\chi_{\alpha }^{2}\left(p\right)$ probabilities associated with the Lagrange test are less than the set critical value α. In this case, a first-order difference filter can correct the autocorrelation and produce a homoscedastic time series [20].

- Normality test

The objective is to ensure that the error terms within the model are independent and identically distributed within the specified model, and therefore make up white noise [21]. This test is backed by the validity of the Jarque-Bera (JB) statistic defined by the equation Eq. (6). The results of this test should be such that JB<5.99 ror a P−value>5░% for the hypothesis of a normal distribution of the error terms within the model to be successful.

(7) $$JB=\left(n/6\right)*\beta_{1}+\left(n/24\right)*{\left(\beta_{2}-3\right)}^{2}$$

$\beta_{1}$ and $\beta_{2}$are respectively coefficients of Skewness and Kurtosis.

- Model stability test

The stability test is performed using the cumulative sum of the recursive residualsφ(t) and the cumulative sum of the squares of the recursive residuals $\overset{\prime }{\varphi }\left(t\right)$ defined by the Eqs. (8) and (9) suggested by Brown et al. [24].

(8) $$\varphi \left(t\right)=\left(n-k\right)\left[\left(\sum_{i=k+1}^{t}{\tilde{\upsilon }}_{j}\right)/\left(\sum_{i=k+1}^{n}{\tilde{\upsilon }}_{j}^{2}\right)\right]$$

(9) $$\overset{\prime }{\varphi }\left(t\right)=\;\left[\left(\sum_{i=k+1}^{t}{\tilde{\upsilon }}_{j}\right)/\left(\sum_{i=k+1}^{n}{\tilde{\upsilon }}_{j}^{2}\right)\right]$$

(*t* = *k* + 1,…, n). The number of variables is k, and the number of observations is n.

The trends in the φ(t) and $\overset{\prime }{\varphi }\left(t\right)$ statistics must be contained within the range defined by the relationship

Eq. (10) for the validation of the long run stability hypothesis of the model specified [24].

(10) $$I=\left[\pm \beta *\left(2t+n-3k\right)/\;\sqrt{\left(n-k\right)}\right]$$

**Fig. 3:**
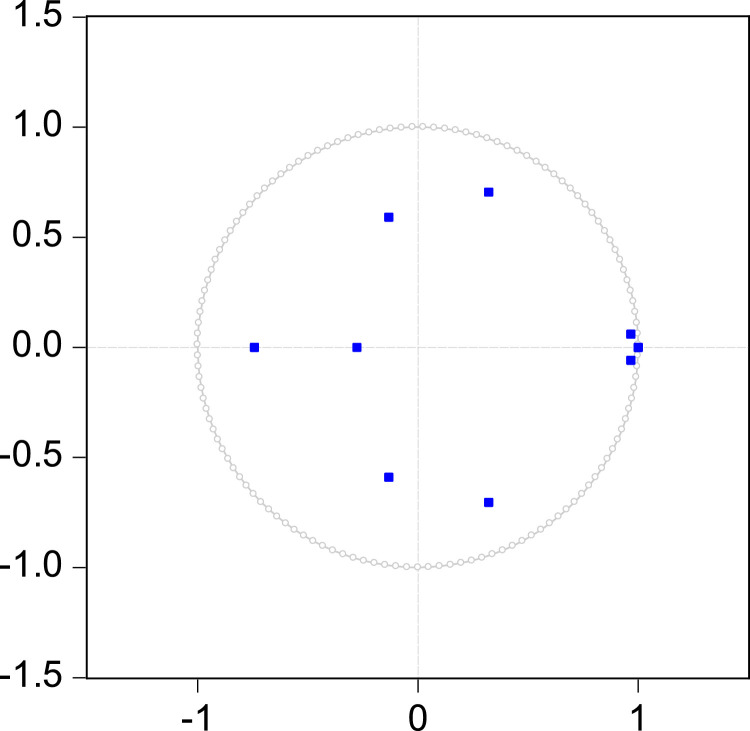
Inverse roots of AR characteristic polynomial [2].

In this relation, β=0.948, at the critical threshold α=5░% [25]. Furthermore, as a final step in analyzing the stability of the ARDL model, it is useful to check all of its inverse roots with respect to the unit cycle. As shown in Fig. 3, all roots denoted by blue dots must be within the unit circle to ensure the stability of the model.

The method described above enables accurate assessment of the explanatory factors of electricity consumption. A case study is presented in the published article, providing an overview of the type of results that can be obtained, and thus facilitating the reproduction of the proposed econometric method. The graphical abstract outlines the sequential steps involved in carrying out the tests needed to rigorously select the explanatory factors of electricity consumption by means of causality analysis.

## Limitations

The econometric methodology described in this paper is limited by its application framework. Indeed, it is prescribed within the framework of selecting the most significant input variables in the context of analyzing fluctuations in electricity consumption. This limitation therefore leaves the door open for future research, particularly in the context of selecting the most significant inputs related to the consumption of petroleum products or various forms of renewable energy (solar energy, wind energy, etc.).

## Ethics statements

***Not applicable***

## Author's Role

**Serge Guefano**: Conceptualization, Data curation, Software,Methodology, Investigation, Preparation, Writing – original draft, Formal analysis, Resources, Validation.

## Supplementary material *and/or* additional information [OPTIONAL]

https://data.mendeley.com/datasets/b7xtffwy4t/1

## Declaration of competing interest

The authors declare that they have no known competing financial interests or personal relationships that could have appeared to influence the work reported in this paper.

## Data Availability

Data will be made available on request.

## References

[1] Chontanawat Jaruwan (2020). Dynamic modelling of causal relationship between energy consumption, CO2 emission, and economic growth in SE Asian countries. Energies.

[2] GUEFANO Serge, BOZORG Mokhtar, CARPITA Mauro (2023). Causality between residential electricity consumption and explanatory factors. Energy Strategy Rev..

[3] GUL Sehrish, ZOU Xiang, HASSAN Che Hashim (2015). Causal nexus between energy consumption and carbon dioxide emission for Malaysia using maximum entropy bootstrap approach. Environ. Sci. Pollut. Res..

[4] FinMath user guide. Augmented Dicked-Fuller (ADF) Tests, 2020.https://rtmath.net/assets/docs/finmath.

[5] FORBES Kevin F., ZAMPELLI Ernest M. (2019). Wind energy, the price of carbon allowances, and CO2 emissions: evidence from Ireland. Energy Policy.

[6] GLYNN, John, PERERA, Nelson, et VERMA, Reetu. Unit root tests and structural breaks: a survey with applications. 2007. https://ro.uow.edu.au/commpapers.

[7] VOGELSANG Timothy J, WAGNER Martin (2013). A fixed-b perspective on the Phillips-Perron unit root tests. Econ Theory.

[8] BOURBONNAIS, Régis. Économétrie-10e éd.: cours et exercices corrigés. Dunod, 2018.

[9] ASUMADU-SARKODIE Samuel, OWUSU Phebe Asantewaa (2016). The relationship between carbon dioxide and agriculture in Ghana: a comparison of VECM and ARDL model. Environ. Sci. Pollut. Res..

[10] HANNINEN, Riitta. The law of one price In United Kingdom soft sawnwood imports-A cointegration approach. Modern time series analysis in forest products markets, 1999, p. 55–68. 10.1007/978-94-011-4772-9-4.

[11] ULLAH Arif, KHAN Dilawar (2020). Testing environmental Kuznets curve hypothesis in the presence of green revolution: a cointegration analysis for Pakistan. Environ. Sci. Pollut. Res..

[12] BARKHORDARI Sajjad, FATTAHI Maryam (2017). Reform of energy prices, energy intensity and technology: a case study of Iran (ARDL approach). Energy Strategy Rev..

[13] ARFANUZZAMAN M.D (2014). The long-run dynamic relationship between broad money supply and the GDP of Bangladesh: a VECM approach. Dev. Ctry. Stud..

[14] Borozan Djula (2013). Exploring the relationship between energy consumption and GDP: evidence from Croatia. Energy Policy.

[15] DOLADO Juan J., GONZALO Jesus, MARMOL Francesc (2003). Cointegration. Companion Theor. Econom..

[16] HUH Kwang-Sook (2011). Steel consumption and economic growth in Korea: long-term and short-term evidence. Resour. Policy.

[17] SAMARGANDI Nahla, SOHAG Kazi (2022). The interaction of finance and innovation for low carbon economy: evidence from Saudi Arabia. Energy Strategy Rev..

[18] MOHSIN Muhammad, NASEEM Sobia, ZIA-UR-REHMAN Muhammad (2023). The crypto-trade volume, GDP, energy use, and environmental degradation sustainability: an analysis of the top 20 crypto-trader countries. Int. J. Finance Econ..

[19] ASLAM Muhammad (2014). Using heteroscedasticity-consistent standard errors for the linear regression model with correlated regressors. Commun. Stat.-Simul. Comput..

[20] BOURBONNAIS Régis, MÉRITET Sophie (2007). The Econometrics of Energy Systems.

[21] PUTUNOI Godfrey K, MUTUKU Cyrus M (2013). Domestic debt and economic growth nexus in Kenya. Curr. Res. J. Econ. Theory.

[22] Mebratu Agumas Alamirew (2023). Mebratu-B-PLC theory implication on developing country’s oxygen of bank. Cogent Econ. Finance.

[23] AMIN Sakib Bin, AL K.A.B.I.R., Foqoruddin, KHAN Farhan (2020). Energy-output nexus in Bangladesh: a two-sector model analysis. Energy Strategy Rev..

[24] Akinlo A.Enisan (2006). The stability of money demand in Nigeria: an autoregressive distributed lag approach. J. Policy Model.

[25] AL-LABADI L., ZAREPOUR M. (2017). Two-sample Kolmogorov-Smirnov test using a bayesian nonparametric approach. Math. Methods Stat..

